# Late-Course Lactate/Albumin Ratio and In-Hospital Mortality in CRRT-Treated Critically Ill Patients: A Retrospective Severity-Adjusted Analysis

**DOI:** 10.3390/jcm15176625

**Published:** 2026-08-27

**Authors:** Hasan Şenay, Mehmet Kürşad Orhan, Mahmut Sami Tutar, Munise Yıldız, Ece Ünüvar Şenay, Muhammed Halit Satıcı, Ahmet Erarkadaş, Betul Kozanhan

**Affiliations:** 1 Department of Intensive Care, Konya City Hospital, 42020 Konya, Turkey; 2 Department of Anaesthesiology and Reanimation, Konya City Hospital, 42020 Konya, Turkey; 3 Department of Pulmonology, Konya Numune Hospital, 42060 Konya, Turkey

**Keywords:** lactate/albumin ratio, continuous renal replacement therapy, CRRT, CVVHDF, APACHE II, SOFA, intensive care, in-hospital mortality, severity adjustment

## Abstract

**Background/Objectives:** The lactate/albumin ratio (LAR) integrates tissue hypoperfusion and nutritional reserve into a single dimensionless index. As a late-course measurement (last available value before the primary outcome), it may reflect terminal metabolic deterioration rather than early prognostic signal. We examined the association of the final LAR with in-hospital mortality after adjustment for validated severity scores (APACHE II, SOFA) in critically ill patients receiving CRRT, and evaluated its incremental value beyond lactate alone. **Methods:** A single-centre retrospective observational study was conducted, including 110 adult patients treated with CRRT/CVVHDF at Konya City Hospital (January 2024–January 2026). Multivariable logistic regression was performed across four hierarchically adjusted models. APACHE II was designated the primary adjustment covariate. ROC analyses compared the final LAR with final lactate and albumin alone. **Results:** In-hospital mortality was 70.9% (78/110); complete paired final lactate and albumin values were available for 109/110 patients (78 non-survivors, 31 survivors). After APACHE II adjustment, each 0.1-unit increase in the late-course LAR remained significantly associated with in-hospital mortality (OR 1.70; 95% CI 1.16–2.49; *p* = 0.007), a finding replicated in the ICU-stay sensitivity cohort (*n* = 100, 73 events; OR 1.63; 95% CI 1.09–2.42; *p* = 0.016). The LAR trajectory (ΔLAR; AUC 0.725) was significantly greater in non-survivors (+0.104 vs. −0.060; *p* < 0.001); however, ΔLAR did not significantly outperform the lactate trajectory alone (Δlactate; AUC 0.706; *p* = 0.212), and lost statistical significance after severity-score adjustment. Furthermore, the association was attenuated and non-significant after SOFA adjustment (OR 1.35; *p* = 0.077), and the final LAR did not provide statistically significant discriminative advantage over lactate alone (AUC 0.768 vs. 0.761; LRT *p* = 0.435). **Conclusions:** The late-course LAR remained significantly associated with mortality after APACHE II adjustment. Three qualifications temper this finding: the association was attenuated after SOFA adjustment, the final LAR did not outperform lactate alone, and the measurement design is susceptible to reverse causality. The LAR trajectory (ΔLAR; AUC 0.725) showed comparable discrimination to the final LAR but did not significantly outperform the lactate trajectory alone (Δlactate; AUC 0.706; *p* = 0.212) and lost statistical significance after severity-score adjustment, so it should likewise be regarded as hypothesis-generating rather than confirmatory; prospective evaluation with time-stamped serial measurements and landmark analyses in multi-centre CRRT cohorts is warranted.

## 1. Introduction

Acute kidney injury (AKI) affects up to 50–60% of critically ill patients, and when CRRT is required, in-hospital mortality may exceed 70% [1,2]. Identifying bedside biomarkers that carry prognostic information independent of established severity scores would complement existing risk-stratification tools in this high-mortality population.

Serum lactate reflects tissue oxygen debt and anaerobic metabolism [3], whereas hypoalbuminaemia signals malnutrition, capillary leak, and reduced hepatic synthetic reserve [4]. The LAR integrates these two dimensions and has shown prognostic value in septic emergency department patients [5], traumatic brain injury [6], and mixed ICU populations [7]. In CRRT-treated patients specifically, only a small number of studies have examined LAR as a prognostic marker [8,9], and none has formally tested whether the LAR association with mortality survives adjustment for established severity scores such as APACHE II or SOFA. To our knowledge, no previous CRRT study has simultaneously evaluated late-course LAR, severity-adjusted prognostic performance, and dynamic LAR trajectory (ΔLAR). This represents a critical gap: CRRT-treated patients exhibit markedly elevated severity scores, and a biomarker association that disappears after severity adjustment provides no incremental information beyond existing risk-stratification tools. Moreover, existing studies evaluated LAR at a single admission time point and did not examine the prognostic value of the dynamic LAR trajectory across the clinical course. Whether the late-course LAR—or its change from the 24 h measurement—provides prognostic information beyond established severity assessment remains unknown.

This study therefore addressed three objectives: (i) to compare clinical and severity characteristics between survivors and non-survivors in CRRT-treated ICU patients; (ii) to examine whether the late-course LAR is associated with mortality after formal adjustment for APACHE II and SOFA, and to quantify any incremental information beyond lactate alone; and (iii) to evaluate the prognostic value of the LAR trajectory (ΔLAR) as a dynamic alternative to a single terminal measurement.

## 2. Materials and Methods

### 2.1. Study Design and Setting

A single-centre retrospective observational study was conducted at the Reanimation 1 ICU (level III) of Konya City Hospital, Konya, Turkey (January 2024–January 2026). Ethics approval was obtained from the KTO Karatay University Faculty of Medicine Non-Drug and Non-Medical Device Research Ethics Committee (decision no. 2026/062; 21 May 2026), in accordance with the Declaration of Helsinki. The requirement for informed consent was waived due to the retrospective and anonymised nature of the study.

### 2.2. Participants

Adults (≥18 years) who received CRRT/CVVHDF with complete APACHE II, SOFA, and final LAR data were included. Exclusion criteria were: age < 18 years; no CRRT; and missing mortality outcome. For patients with multiple admissions, only the first was included. ICU discharge occurred either through in-ICU death or transfer to a general ward; patients transferred to the ward were followed for the primary outcome of in-hospital mortality, so patients who left the ICU alive but subsequently died during the same hospital admission were classified as non-survivors, while only patients discharged alive from the hospital were classified as survivors.

### 2.3. Data Collection

Data extracted from electronic records included: age, sex, admission diagnosis, MV and vasopressor need, ICU and hospital LOS, MV and CRRT duration, and in-hospital mortality. APACHE II and SOFA scores were recorded at ICU admission. Laboratory parameters were collected at (a) 24 h after ICU admission and (b) the last available measurement before the primary outcome event (“final” values): serum lactate, albumin, blood urea nitrogen (BUN), and creatinine. Serum lactate was measured from arterial blood gas samples throughout the study period. Laboratory parameters were obtained through once-daily routine morning blood sampling as per standard unit protocol; the “final” values therefore correspond to the last routine morning measurement obtained on the calendar day preceding, or the day of, the primary outcome event. Exact clock-time intervals between this measurement and the outcome event were not captured in the electronic record.

### 2.4. Severity Scores and Adjustment Strategy

APACHE II (range 0–71) and SOFA (range 0–24) were both recorded at ICU admission and available for all 110 patients. Both scores therefore reflect early, admission-phase illness severity and are temporally distinct from the final LAR, which was measured at the last available time point before the outcome event. APACHE II showed adequate between-group overlap for regression. SOFA demonstrated a narrow range in survivors (median 8 [IQR 8–8]), suggesting a ceiling effect in the survivor group. Model B (LAR + APACHE II) was designated the primary model; Models C and D, which include SOFA, are presented as sensitivity analyses.

### 2.5. Final LAR Definition and Timing

LAR = serum lactate (mmol/L) ÷ serum albumin (g/L). The “final LAR” was the last available paired measurement before the primary outcome event, drawn from the routine once-daily morning sampling protocol described in Section 2.3. Because sampling occurred once daily rather than continuously or on an event-triggered basis, the interval between this measurement and the outcome event could range from a few hours (e.g., death occurring shortly after morning sampling) up to approximately 24 h (e.g., death occurring late the following day, with no interim sample drawn). No minimum measurement-to-outcome interval was imposed, and exact clock-time timestamps were not available in the electronic record, precluding a formal landmark analysis. This routine-sampling structure, rather than incomplete documentation alone, is an inherent limitation of retrospective ICU datasets and introduces susceptibility to reverse causality, particularly for non-survivors in whom the final measurement may reflect terminal physiology. The final LAR should not be interpreted as an early predictive biomarker; it reflects a late-course metabolic state that may be susceptible to reverse causality, and its clinical utility requires prospective validation with standardised measurement timing. A sensitivity analysis using patients with ICU stay ≥ 2 days was performed to examine robustness.

### 2.6. Statistical Analysis

IBM SPSS Statistics, version 26.0 (IBM Corp., Armonk, NY, USA). Normality: Shapiro–Wilk. Continuous: mean ± SD or median (IQR); categorical: *n* (%). Between-group: Mann–Whitney U or independent *t*-test; chi-square or Fisher exact. Variance inflation factors (VIFs) were calculated to quantify multicollinearity between regression covariates.

Four multivariable logistic regression models were constructed using an a priori hierarchical adjustment strateg . The final LAR was the primary exposure variable and was entered as a continuous variable (per 0.1-unit increase) in all regression models. ROC analysis with AUC (95% CI, 2000-replicate bootstrap) was performed for the final LAR, ΔLAR, final lactate alone, and final albumin alone; APACHE II and SOFA AUCs are reported descriptively. Pairwise AUC comparisons used the bootstrap method. The Youden index was used to identify the optimal cut-off. All analyses were performed at a significance threshold of *p* < 0.05 (two-tailed).

## 3. Results

### 3.1. Patient Characteristics and Severity Scores

One hundred and ten patients formed the analytic cohort (in-hospital mortality 70.9%, *n* = 78; survivors 29.1%, *n* = 32). Both admission APACHE II (29 [IQR 22–34] vs. 16 [IQR 12–20]; *p* < 0.001) and admission SOFA (13 [IQR 11–15] vs. 8 [IQR 8–8]; *p* < 0.001) were markedly higher in non-survivors. Groups did not differ significantly in sex (*p* = 0.620) or any individual admission condition; age showed a borderline non-significant trend towards being higher in non-survivors (60 ± 21 vs. 68 ± 16 years; *p* = 0.070). Mechanical ventilation was required by all non-survivors (100%) versus 71.9% of survivors (*p* < 0.001). MV duration was significantly longer in non-survivors (*p* = 0.030). CRRT duration (*p* = 0.661) and ICU LOS (*p* = 0.313) were similar between groups. Admission BUN (*p* < 0.001) and admission creatinine (*p* = 0.002) were higher in non-survivors. Final BUN was significantly higher in non-survivors (*p* = 0.037), whereas final creatinine did not differ significantly between groups (*p* = 0.096) (Table 1).

### 3.2. Lactate, Albumin, and Final LAR

Of 110 patients, 109 had paired final lactate and albumin measurements available for LAR calculation (78 non-survivors, 31 survivors); one survivor had a missing lactate value at both the 24 h and final time points (albumin was available for this patient at both time points) and was excluded from lactate- and LAR-based analyses. 24 h albumin was available for all 110 patients; 24 h lactate and 24 h LAR were available for 109. The 24 h lactate (*p* = 0.070), 24 h albumin (*p* = 0.668), and 24 h LAR (*p* = 0.196) did not differ significantly at CRRT initiation. In contrast, the final LAR was markedly higher in non-survivors (0.37 [IQR 0.15–0.62] vs. 0.09 [0.07–0.18]; *p* < 0.001), driven by substantially higher final lactate (8.8 [3.8–13.8] vs. 2.6 [1.6–4.9] mmol/L; *p* < 0.001). Final albumin was significantly lower in non-survivors (24.0 [21.3–27.0] vs. 27.0 [24.0–30.5] g/L; *p* = 0.017). The change in LAR from 24 h to the final measurement (ΔLAR) was available for 109 patients (78 non-survivors and 31 survivors) and was significantly greater in non-survivors (+0.10 [IQR −0.01–0.32] vs. −0.06 [−0.12–0.08]; *p* < 0.001), with an AUC of 0.725 (95% CI 0.616–0.824).

### 3.3. Multivariable Logistic Regression and Sensitivity Analysis

Results across all four models and the sensitivity analysis are presented in Table 2. In the unadjusted model (Model A), each 0.1-unit increase in final LAR was significantly associated with mortality (OR 1.58; 95% CI 1.22–2.04; *p* < 0.001; McFadden R^2^ = 0.157).

In the primary severity-adjusted model (Model B: continuous LAR + APACHE II), the final LAR remained significantly associated with mortality after APACHE II adjustment (OR 1.70 per 0.1-unit increase; 95% CI 1.16–2.49; *p* = 0.007), with APACHE II also significant (OR 1.51 per point; 95% CI 1.23–1.85; *p* < 0.001; McFadden R^2^ = 0.606).

When admission SOFA was added (Model C), the LAR association was attenuated (OR 1.35 per 0.1-unit; 95% CI 0.97–1.87; *p* = 0.077) while SOFA remained strongly associated (OR 2.93 per point; 95% CI 1.86–4.60; *p* < 0.001; McFadden R^2^ = 0.627). When both APACHE II and SOFA were included (Model D), the LAR OR further attenuated (OR 1.34; 95% CI 0.89–2.04; *p* = 0.164). The sensitivity analysis (ICU stay ≥ 2 days; *n* = 100, events = 73) was consistent with the primary analysis: the final LAR remained independently associated after APACHE II adjustment (OR 1.63 per 0.1-unit; 95% CI 1.09–2.42; *p* = 0.016) and was attenuated after SOFA adjustment (OR 1.29; 95% CI 0.93–1.78; *p* = 0.123).

### 3.4. ROC Analysis

**Figure 1:**
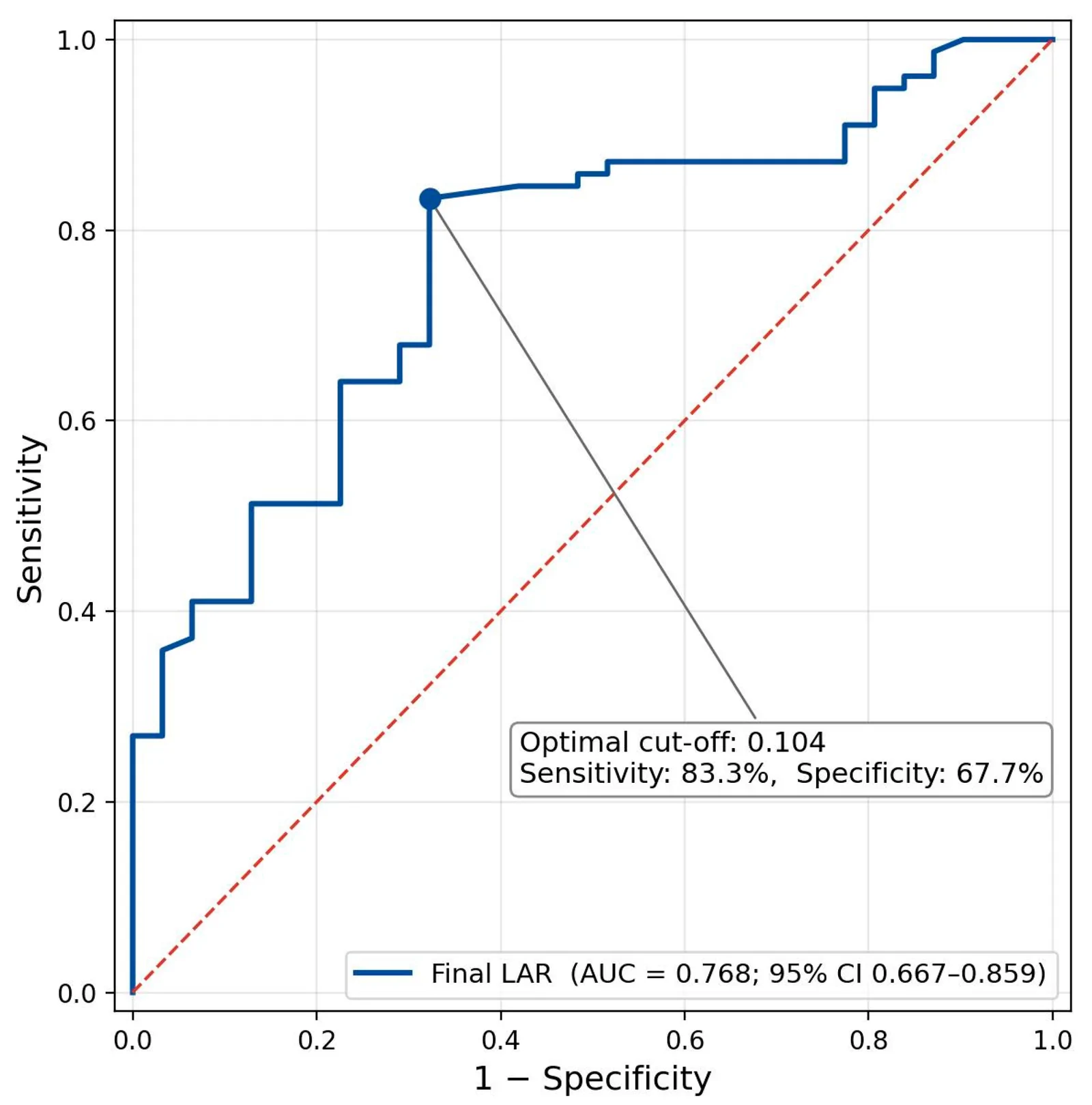
Receiver operating characteristic (ROC) curve of the final lactate/albumin ratio (LAR) for association with in-hospital mortality in CRRT-treated ICU patients (*n* = 109). The area under the curve (AUC) was 0.768 (95% CI 0.667–0.859). The Youden-optimal cut-off was data-derived and exploratory (0.104), corresponding to a sensitivity of 83.3% and specificity of 67.7%; it should not be used for bedside decision-making without prospective validation. The diagonal line represents no discrimination.

In the 109 patients with complete paired final lactate and albumin data, the final LAR (AUC 0.768; 95% CI 0.667–0.859) numerically exceeded final lactate alone (AUC 0.761; 95% CI 0.663–0.854; ΔAUC +0.007; 95% CI −0.017 to +0.032; *p* = 0.555) and significantly exceeded final albumin alone (AUC 0.634; 95% CI 0.507–0.751; ΔAUC +0.134; 95% CI +0.020 to +0.245; *p* = 0.019). A likelihood ratio test on a nested model (final lactate alone vs. final lactate + final albumin) confirmed that adding albumin does not provide statistically significant discrimination beyond lactate alone (χ^2^ = 0.61; *p* = 0.435). The ΔLAR yielded an AUC of 0.725 (95% CI 0.616–0.824), numerically but not significantly higher than the AUC of the lactate trajectory alone (Δlactate = final lactate − 24 h lactate; AUC 0.706, 95% CI 0.591–0.806; ΔAUC +0.020, 95% CI −0.011 to +0.051; *p* = 0.212), indicating that ΔLAR does not provide statistically significant incremental discrimination over the lactate trajectory alone. A likelihood ratio test on a nested model (Δlactate alone vs. Δlactate + Δalbumin) was consistent with this (χ^2^ = 2.83; *p* = 0.092). Admission SOFA (AUC 0.950) and APACHE II (AUC 0.924) showed superior discrimination. The Youden index identified an optimal final LAR cut-off of 0.104 (sensitivity 83.3%, specificity 67.7%) (Table 3, Figure 1).

## 4. Discussion

### 4.1. Principal Finding

Three findings characterise this study. First, the final LAR remained significantly associated with mortality after APACHE II adjustment and this finding was reproduced in the ICU-stay sensitivity cohort. Second, after admission SOFA adjustment, the LAR association was attenuated and non-significant (OR 1.35; *p* = 0.077), suggesting limited incremental value beyond early organ failure burden as captured by admission SOFA. Third, the LAR did not provide statistically significant discrimination beyond lactate alone (AUC 0.768 vs. 0.761; LRT *p* = 0.435), indicating that lactate appears to capture most of the discriminatory signal within this composite index. Taken together, these results are consistent with a late-course metabolic severity marker rather than a stand-alone early predictive biomarker. Notably, the LAR trajectory (ΔLAR; AUC 0.725) performed comparably to the final LAR, suggesting that the direction of metabolic change across the CRRT course may carry at least as much prognostic information as any single terminal measurement; however, as detailed in Section 4.6, ΔLAR did not significantly outperform the lactate trajectory alone and did not retain significance after severity-score adjustment, so this trajectory signal should also be regarded as hypothesis-generating.

### 4.2. APACHE II vs. SOFA: Mechanistic Interpretation

APACHE II captures a static admission cross-section of severity. The final LAR, measured later in the clinical course, may reflect post-admission metabolic trajectory not yet visible at admission, which could explain why the LAR OR increases rather than decreases after APACHE II adjustment. This pattern has been described as “negative confounding” [10], though several alternative explanations are plausible, including treatment intensity differences or simple statistical behaviour in small samples. In the absence of treatment protocol data, any causal interpretation of this OR amplification is speculative and should not be over-interpreted.

Admission SOFA captures multisystem organ dysfunction across six physiological domains [11]. The final LAR shares physiological overlap with several of these: lactate reflects cardiovascular and hepatic perfusion, while albumin captures hepatic synthetic reserve and capillary integrity. The VIF values remained low (<1.4) in all models, indicating that collinearity does not explain the attenuation numerically. These findings suggest that final LAR should not be interpreted as an independent marker beyond admission SOFA in this dataset, and future biomarker studies in CRRT populations should test candidate markers a priori against both admission and serial SOFA [12].

### 4.3. Timing Bias and Methodological Caution

The final LAR should not be interpreted as an early warning biomarker. Operationally, the “final” measurement was defined as the last available paired lactate and albumin value before the primary outcome event, with no minimum measurement-to-outcome interval imposed and no individual timestamps available. This design introduces susceptibility to reverse causality: for patients who died shortly after admission, the final LAR may reflect terminal metabolic collapse rather than an upstream prognostic signal. Studies of lactate kinetics in critically ill and dying patients suggest that serial lactate measurements carry important prognostic information [13], and that lactate may rise substantially within the final hours of life as hepatic clearance fails [14]. The sensitivity analysis restricted to ICU stay ≥2 days produced consistent results (OR 1.63; *p* = 0.016), offering partial reassurance; however, this proxy cannot substitute for a formal landmark analysis [15].

### 4.4. Lactate as the Dominant Driver

The near-identical AUC of the final LAR and final lactate alone (0.768 vs. 0.761; LRT *p* = 0.435) indicates that lactate appears to capture most of the discriminatory signal provided by the composite index in this cohort, with albumin contributing limited additional discrimination. This finding is consistent with the established primacy of lactate as a mortality predictor in critical illness [3,16] and with international guidelines that recommend lactate-guided resuscitation [17]. The biological basis is coherent: in critically ill patients, the conditions that elevate lactate—sepsis, hepatic failure, prolonged ICU stay—simultaneously suppress albumin [5,7], so the two components are not informationally independent.

### 4.5. Comparison with the Prior Literature

Previous studies have consistently reported an association between an elevated LAR and adverse outcomes across heterogeneous critically ill populations. Bou Chebl et al. reported that admission LAR was associated with in-hospital mortality among septic patients presenting to the emergency department [5], while Gharipour et al. similarly demonstrated prognostic value in a mixed critically ill population [7]. Wang et al. extended these findings to patients with moderate-to-severe traumatic brain injury [6], further supporting the potential value of LAR as an integrated marker of circulatory stress and physiological reserve.

More recent studies have specifically examined populations with kidney dysfunction. In a multi-centre observational analysis of 4666 critically ill patients with AKI, an increasing LAR was independently associated with both hospital and ICU mortality after multivariable adjustment, supporting the relevance of this index in patients with impaired renal function [18]. More directly relevant to the present cohort, Liu et al. analysed 703 patients with AKI undergoing CRRT and demonstrated a nonlinear association between LAR and both 28-day and 360-day mortality; patients in the highest LAR category had significantly greater mortality after adjustment for multiple covariates [8].

Recent studies in sepsis-associated AKI have provided similar findings. In an eICU-based cohort of 1855 patients, higher LAR was independently associated with 28-day mortality, with evidence of a nonlinear exposure–response relationship [19]. A separate MIMIC-IV analysis involving 3589 patients with sepsis-associated AKI also demonstrated progressively greater in-hospital, 30-day, and 90-day mortality across increasing LAR categories [20]. He et al. likewise reported an association between LAR and 28-day mortality in patients with sepsis-associated AKI [9].

Importantly, however, the timing and interpretation of LAR in these studies differ fundamentally from those in the present analysis. Most previous investigations evaluated LAR at ICU admission or during an early predefined time window, whereas our primary exposure was the last routinely available LAR before the outcome event. Consequently, direct comparison of effect estimates or ROC performance should be made cautiously. Admission LAR may function as an early risk-stratification marker, whereas the late-course LAR examined here more likely reflects evolving metabolic deterioration during critical illness and is inherently more susceptible to reverse causality.

This temporal distinction may also explain an important divergence from previous reports. Although earlier studies generally found LAR to retain an independent association with mortality after multivariable adjustment, the association in our cohort was attenuated and became non-significant after adjustment for admission SOFA. Furthermore, final LAR did not significantly outperform final lactate alone. These findings suggest that, in CRRT-treated patients assessed late in their clinical course, much of the apparent prognostic information carried by LAR may reflect the lactate component and the underlying burden of organ failure rather than a distinct incremental signal provided by albumin.

The importance of assessing incremental value beyond established severity measures is supported by other biomarker research in CRRT populations. For example, a multi-centre study of 3995 critically ill patients requiring CRRT found that the C-reactive protein-to-albumin ratio was associated with mortality and improved discrimination when combined with APACHE II or SOFA [21]. Such findings highlight that the clinical usefulness of a composite biomarker should not be judged solely by its unadjusted association with mortality, but also by whether it adds information beyond validated severity scores.

Taken together, the available literature supports an association between elevated LAR and mortality in critically ill and AKI populations, including patients receiving CRRT. The present study extends this evidence by examining a late-course measurement, directly comparing LAR with lactate alone, formally adjusting for both APACHE II and SOFA, and evaluating longitudinal change in LAR. At the same time, our findings indicate that the clinical interpretation of late-course LAR should be more cautious than that of admission LAR and should focus on association with evolving metabolic severity rather than early mortality prediction.

### 4.6. Prognostic Implications of the Dynamic LAR Trajectory

The ΔLAR (final LAR minus 24 h LAR) was significantly greater in non-survivors (+0.10 [IQR −0.01–0.32] vs. −0.06 [−0.12 to +0.08]; *p* < 0.001) and yielded an AUC of 0.725 (95% CI 0.616–0.824), comparable to the final LAR (AUC 0.768) and substantially superior to final albumin alone (AUC 0.634). Unlike a terminal static value, ΔLAR captures directional metabolic worsening across the CRRT course. A positive ΔLAR requires both a stable or low baseline measurement and subsequent deterioration, making it intrinsically less susceptible to the reverse causality that affects a single peri-death LAR reading. In patients in whom the final LAR is elevated purely due to terminal physiology, the 24 h LAR is typically within the reference range; a large positive ΔLAR may therefore better distinguish genuine progressive metabolic deterioration from isolated peri-death hyperlactataemia [13,14]. To determine whether this trajectory-level composite index adds value beyond the lactate trajectory alone, we compared ΔLAR with Δlactate (final lactate minus 24 h lactate). The AUC for ΔLAR (0.725) was numerically but not significantly higher than that for Δlactate alone (0.706; ΔAUC +0.020; 95% CI −0.011 to +0.051; *p* = 0.212), and a nested likelihood ratio test (Δlactate alone vs. Δlactate + Δalbumin) was consistent with this (χ^2^ = 2.83; *p* = 0.092). Therefore, as with the final (static) LAR, the dynamic composite index does not provide statistically significant incremental discrimination over its lactate component alone in this cohort—although, for the static comparison, this negative finding should be interpreted with caution given the modest sample size. These properties position ΔLAR as a candidate for prospective validation with standardised, time-stamped serial measurements, predefined landmark time points, and formal trajectory modelling in multi-centre CRRT cohorts. In exploratory multivariable analysis, each 0.1-unit increase in ΔLAR was significantly associated with mortality in the unadjusted model (OR 1.27; 95% CI 1.09–1.48; *p* = 0.003), but this association did not persist after adjustment for APACHE II (OR 1.19; 95% CI 0.94–1.50; *p* = 0.146) or SOFA (OR 1.36; 95% CI 0.98–1.89; *p* = 0.063). This pattern mirrors that of the final LAR itself and suggests that, despite its favourable discrimination on ROC analysis (AUC 0.725), ΔLAR does not yet demonstrate independent incremental value beyond established severity scores in this cohort; formal confirmation will require a larger, prospectively timed dataset.

### 4.7. CRRT-Specific Biomarker Considerations

CRRT introduces two circuit-related confounders. First, lactate-buffered replacement fluids may elevate serum lactate by 0.5–2.0 mmol/L, particularly in patients with impaired hepatic clearance, inflating the LAR in a manner that reflects circuit design rather than tissue hypoperfusion [22,23]. Second, filter-related albumin loss can progressively lower serum albumin, artificially elevating the LAR independently of nutritional status [24,25]. Neither effect could be corrected for in this study, as replacement fluid composition, filter type, and albumin supplementation were not recorded.

### 4.8. Limitations

The following limitations should be considered: (1) Retrospective single-centre design; *n* = 110 limits power. (2) SOFA survivor range was narrow (7–8), reflecting limited discrimination within that group. (3) Measurement timing: laboratory values were obtained through once-daily routine morning sampling rather than continuous or event-triggered monitoring; the final LAR therefore corresponds to an interval of approximately a few hours up to 24 h before the outcome event, but exact clock-time timestamps were not available, precluding a formal landmark analysis. (4) CRRT-related and general fluid-management confounders: data on replacement fluid composition (lactate- versus bicarbonate-buffered), filter characteristics, CRRT dose, and albumin supplementation were not available; more broadly, no data were available on the volume, type, or timing of intravenous fluids or vasopressor infusions administered during the ICU stay, any of which could independently affect serum lactate and albumin concentrations and therefore the LAR, independent of underlying tissue perfusion. (5) Cohort heterogeneity: sepsis, AKI, hypovolemic shock, and multi-organ failure were pooled; subgroup analyses were underpowered. (6) With 78 events in the primary cohort of 109 patients, effect estimates should be interpreted cautiously.

## 5. Conclusions

In CRRT-treated ICU patients, the late-course LAR showed a significant but limited association with in-hospital mortality. The key findings were:

(1) The late-course LAR should not be interpreted as an early predictive biomarker. Because the final measurement was obtained through once-daily routine sampling without a standardised measurement-to-outcome interval, the observed association is inherently susceptible to reverse causality and may reflect late metabolic deterioration.
(2) After APACHE II adjustment, each 0.1-unit increase in the final LAR remained significantly associated with in-hospital mortality (OR 1.70; 95% CI 1.16–2.49; *p* = 0.007), and this finding was replicated in the ICU-stay sensitivity cohort (OR 1.63; *p* = 0.016).
(3) In contrast, the association was attenuated and became non-significant after adjustment for SOFA (OR 1.35; *p* = 0.077), indicating that the final LAR did not demonstrate independent incremental value beyond the organ failure burden captured by SOFA in this cohort.
(4) The final LAR did not provide statistically significant discrimination beyond final lactate alone (AUC 0.768 vs. 0.761; *p* = 0.435), suggesting that most of the discriminatory information carried by the composite index was driven by lactate, with limited incremental contribution from albumin.
(5) The ΔLAR trajectory (AUC 0.725) was significantly associated with mortality in unadjusted analysis (OR 1.27 per 0.1-unit increase; *p* = 0.003) but did not retain statistical significance after adjustment for APACHE II or SOFA; ΔLAR also did not provide statistically significant discrimination beyond the lactate trajectory alone (Δlactate, AUC 0.706; *p* = 0.212), mirroring the pattern seen for the static final LAR. These findings support prospective evaluation of serial LAR measurements using standardised, time-stamped sampling and predefined landmark analyses in larger multi-centre CRRT cohorts.

## Figures and Tables

**Table 1:** Demographic, severity, clinical, and laboratory characteristics by in-hospital outcome.

*p* Value	Non-Survivors (*n* = 78)	Survivors (*n* = 32)	Variable
0.070	68 ± 16	60 ± 21	Age, years
0.620	30 (38.5)	10 (31.3)	Female sex, *n* (%)
<0.001	29 (22–34)	16 (12–20)	APACHE II score
<0.001	13 (11–15)	8 (8–8)	SOFA score
0.782			Admission conditions, *n* (%)
	48 (61.5)	14 (45.2)	Acute kidney injury
	27 (34.6)	7 (22.6)	Hypovolemic shock
	13 (16.7)	4 (12.9)	Multi-organ failure
	13 (16.7)	6 (19.4)	Sepsis ± AKI
0.070	4.9 (2.5–8.2)	3.4 (2.0–5.2)	24 h lactate, mmol/L
0.668	25.0 (23.0–29.0)	27.0 (21.8–31.0)	24 h albumin, g/L
0.196	0.21 (0.10–0.34)	0.14 (0.08–0.19)	24 h LAR
<0.001	78 (100.0)	23 (71.9)	Mechanical ventilation, *n* (%)
0.030	7.0 (3.0–15.5)	4.5 (0.0–10.2)	MV duration, days
0.661	3.0 (2.0–5.0)	3.0 (2.0–4.0)	CRRT duration, days
0.313	9.5 (3.0–18.0)	7.0 (4.0–10.5)	ICU LOS, days
<0.001	64.5 (40.0–91.8)	32.0 (24.2–43.8)	Admission BUN, mg/dL
0.002	2.70 (1.71–3.91)	1.80 (0.81–2.84)	Admission creatinine, mg/dL
<0.001	8.8 (3.8–13.8)	2.6 (1.6–4.9)	Final lactate, mmol/L
0.017	24.0 (21.3–27.0)	27.0 (24.0–30.5)	Final albumin, g/L
<0.001	0.37 (0.15–0.62)	0.09 (0.07–0.18)	Final LAR
0.037	29.0 (17.2–55.8)	22.5 (10.8–28.5)	Final BUN, mg/dL
0.096	1.30 (0.70–2.51)	0.77 (0.58–1.73)	Final creatinine, mg/dL

Data: mean ± SD, median (IQR), or *n* (%). Denominators are *n* = 32 (survivors)/*n* = 78 (non-survivors) for all rows except 24 h lactate, 24 h LAR, final lactate, and final LAR, for which survivor *n* = 31 (one survivor had a missing lactate value at both the 24 h and final time points; all other laboratory rows, including final albumin, final BUN, and final creatinine, had no missing values). AKI, acute kidney injury; APACHE II, Acute Physiology and Chronic Health Evaluation II; CRRT, continuous renal replacement therapy; ICU, intensive care unit; LAR, lactate/albumin ratio; MV, mechanical ventilation; BUN, blood urea nitrogen; SOFA, Sequential Organ Failure Assessment.

**Table 2:** Multivariable logistic regression: association of the final LAR (continuous, per 0.1-unit increase) with in-hospital mortality—primary analysis and sensitivity analysis.

*n*/Events	*p* Value	95% CI	OR	Variable
				Model A—Unadjusted
109/78	<0.001	1.22–2.04	1.58	Final LAR (per 0.1-unit increase)
				Model B—Adjusted for APACHE II (★ Primary model)
109/78	0.007	1.16–2.49	1.70	Final LAR (per 0.1-unit increase)
	<0.001	1.23–1.85	1.51	APACHE II, per point
				Model C—Adjusted for SOFA
109/78	0.077	0.97–1.87	1.35	Final LAR (per 0.1-unit increase)
	<0.001	1.86–4.60	2.93	SOFA, per point
				Model D—Adjusted for APACHE II + SOFA
109/78	0.164	0.89–2.04	1.34	Final LAR (per 0.1-unit increase)
				Sensitivity Analysis—ICU stay ≥ 2 days (*n* = 100/events = 73)
100/73	0.002	1.16–1.96	1.51	Final LAR (per 0.1-unit), unadjusted
100/73	0.016	1.09–2.42	1.63	Final LAR (per 0.1-unit), + APACHE II

OR, odds ratio; CI, confidence interval; LAR, lactate/albumin ratio; APACHE II, Acute Physiology And Chronic Health Evaluation II; SOFA, Sequential Organ Failure Assessment. ★ Primary model. McFadden R^2^ (Model B) = 0.606; VIF: LAR = 1.06, APACHE II = 1.06.

**Table 3:** ROC analysis: discriminative performance for in-hospital mortality.

*p*	Specificity (%)	Sensitivity (%)	Optimal Cut-Off	95% CI †	AUC	Marker
<0.001	—	—	—	0.666–0.853	0.761	Final lactate alone
<0.001	—	—	—	0.513–0.756	0.634	Final albumin alone
<0.001	67.7	83.3	0.104 *	0.667–0.859	0.768	Final LAR (primary analysis)
<0.001	70.4	82.2	0.104	—	0.751	Final LAR (sensitivity, ≥2-day ICU stay)
<0.001	—	—	—	0.616–0.824	0.725	ΔLAR (dynamic trajectory)
<0.001	—	—	—	0.591–0.806	0.706	Δlactate (lactate trajectory)
<0.001	—	—	—	—	0.924	Admission APACHE II ‡
<0.001	—	—	—	—	0.950	Admission SOFA ‡

AUC, area under the curve; CI, confidence interval; LAR, lactate/albumin ratio; APACHE II, Acute Physiology and Chronic Health Evaluation II; SOFA, Sequential Organ Failure Assessment. Primary analysis *n* = 109 (all markers except sensitivity LAR); sensitivity cohort *n* = 100. * Optimal cut-off by Youden index (data-derived exploratory threshold; prospective validation required). † Bootstrap 2000 replicates. ‡ AUC reported descriptively; cut-off, sensitivity, and specificity not derived (severity scores not biomarker candidates). Pairwise comparisons: Final LAR vs. lactate: ΔAUC = +0.007 (95% CI −0.017–+0.032), *p*=0.555; Final LAR vs. albumin: ΔAUC = +0.134 (95% CI +0.020–+0.245), *p* = 0.019. ΔLAR vs. Δlactate: ΔAUC = +0.020 (95% CI −0.011–+0.051), *p* = 0.212.

## Data Availability

Data are available from the corresponding author on reasonable request.

## Abbreviations

The following abbreviations are used in this manuscript:

AKI	Acute kidney injury
APACHE II	Acute Physiology And Chronic Health Evaluation II
AUC	Area under the curve
BUN	Blood urea nitrogen
CI	Confidence interval
CRRT	Continuous renal replacement therapy
CVVHDF	Continuous veno-venous haemodiafiltration
ICU	Intensive care unit
IQR	Interquartile range
LAR	Lactate/albumin ratio
LOS	Length of stay
MV	Mechanical ventilation
OR	Odds ratio
ROC	Receiver operating characteristic
SOFA	Sequential Organ Failure Assessment
VIF	Variance inflation factor
